# Screening for Periodontitis Using Blood Biomarkers and Demographic Data: A Machine Learning Study

**DOI:** 10.3290/j.ohpd.c_2684

**Published:** 2026-05-20

**Authors:** Seongwon Choi, Daniel Oh, Rami Rashed, Rami Hemadeh, Yoon Jeong Kim, Udochukwu Oyoyo

**Affiliations:** a #Seongwon Choi Dentist and Endodontic resident, Advanced Dental Education Program in Endodontics, School of Dentistry, Loma Linda University, Loma Linda, CA, USA. Conceived the study, performed the analyses, and drafted the manuscript.; b #Daniel Oh Researcher, Department of Statistics and Data Science, University of California, Los Angeles, CA, USA. Conceived the study, performed the analyses, and drafted the manuscript.; c Rami Rashed Dentist and Periodontal Resident, Advanced Dental Education Program in Periodontics, School of Dentistry, Loma Linda University, Loma Linda, CA, USA. Conceived the study, performed the analyses, drafted the manuscript, assisted with literature review, data interpretation, and manuscript revisions.; d Rami Hemadeh Researcher, Department of Psychology, University of California, Los Angeles, CA, USA. Assisted with literature review, data interpretation, and manuscript revisions.; e Yoon Jeong Kim Professor, Program director, Advanced Dental Education Program in Periodontics, School of Dentistry, Loma Linda University, Loma Linda, CA, USA. Contributed to statistical input and manuscript revisions.; f Udochukwu Oyoyo Assistant Professor, Department of Dental Education Services, School of Dentistry, Loma Linda University, Loma Linda, CA, USA. Supervised the project, provided critical revisions, and approved the final version.

**Keywords:** blood biomarkers, machine learning, NHANES, periodontitis, prevention, screening

## Abstract

**Purpose:**

Periodontitis is a common chronic disease associated with systemic conditions such as diabetes and cardiovascular disease. Diagnosis typically relies on dental examinations and radiographs, which may be underutilised by individuals who avoid, delay, or lack dental care. This study evaluated the potential of routine blood biomarkers and demographic data for screening moderate-to-severe periodontitis.

**Methods and Materials:**

Data were obtained from the National Health and Nutrition Examination Survey (NHANES) 2011–2012 adults aged ≥ 30 years (N = 3,338). Periodontal status was classified using CDC/AAP definitions. An XGBoost classifier was trained on 77 features spanning demographics, complete blood count, glycemic and hemo-globin markers, heavy metals, and additional biochemical assays. Model performance was assessed with Accuracy, Precision, Recall, and F1 score. SHapley Additive exPlanations (SHAP) were used for interpretability. Subgroup analyses were conducted by age and gender.

**Results:**

The model achieved 61.1% Accuracy, 57.4% Precision, 94.6% Recall, and an F1 score of 71.5%. Performance was higher in males (F1 = 78.2) than in females (F1 = 63.9%). SHAP identified age, gender, blood cadmium, blood lead, and glycohaemoglobin (HbA1c) as top predictors.

**Conclusion:**

Routine blood biomarkers and demographics can be leveraged for non-dental screening of periodontitis, offering a feasible preventive strategy in primary care. The model achieved high recall, minimising false negatives, and identified biologically plausible predictors. This approach may help flag at-risk individuals, particularly in underserved populations, and support integration of oral health into general medical care.

Periodontitis is a chronic inflammatory disease that destroys periodontal connective tissue and bone, leading to tooth mobility and loss. It affects approximately 50% of adults over 30 in the United States and is a major global oral health issue. Beyond oral consequences, periodontitis has been shown to be linked to a variety of systemic conditions like type 2 diabetes, cardiovascular disease, chronic kidney disease, and adverse pregnancy outcomes.^18^

Although early detection of periodontitis is important, it is often hindered by the high cost of routine dental visits and diagnostic procedures.^26^ As a result, many individuals avoid getting care until the disease has progressed. Our machine learning model addresses this gap by using accessible, non-invasive data, such as complete blood count (CBC) and demographic variables, to screen for moderate-to-severe disease risk. This approach highlights a potential preventive tool that could be integrated into routine medical care, particularly benefiting populations with limited access to dental services.

Diagnosis traditionally relies on periodontal probing and radiographic evaluation – procedures requiring trained dental professionals, which may be unavailable or underutilised by individuals who delay dental care due to cost, dental anxiety, or neglect.^14^ In contrast, medical laboratory tests such as CBC, metabolic panels, and toxicology screens are routinely performed during general health assessments, creating a unique opportunity to explore whether systemic biomarkers may be leveraged for non-dental periodontal risk screening.

Growing recent evidence suggests that systemic biomarkers – including metabolic, inflammatory, and environmental indicators – may reflect or even contribute to periodontal disease processes. For example, elevated fasting glucose and haemoglobin A1c levels are associated with impaired wound healing, increased advanced glycation end-product formation, and amplified periodontal tissue destruction.^15^ Inflammatory biomarkers such as white blood cell count and red cell distribution width have also been shown to correlate with periodontitis severity, possibly reflecting shared immunoinflammatory pathways.^6^ Together, these findings underscore the biological significance of systemic biomarkers in the development and progression of periodontal disease.

More recently, environmental toxins, particularly heavy metals such as cadmium and lead, have emerged as additional contributors to periodontal risk.^10^ These toxicants accumulate in soft tissues, disrupt immune homeostasis, and promote oxidative stress – factors believed to exacerbate periodontal inflammation and bone loss. Epidemiological studies have further shown that heavy metal exposure is associated with elevated levels of systemic inflammatory biomarkers.^10,19^ Yet despite these established associations, their predictive utility in a screening context remains underexplored.

While several studies have examined associations between individual biomarkers and periodontitis, few have leveraged the combination of these markers for predictive modelling using interpretable machine learning. Our study aims to explore the potential of systemic and demographic features in modelling periodontitis severity. This can potentially offer valuable insights into their relative importance and predictive utility. To address this gap, we developed a machine learning model to predict moderate-to-severe periodontitis using 77 input features from the National Health and Nutrition Examination Survey (NHANES) (N = 3,338). These features were grouped into six main categories: (1) Demographic variables (age and gender); (2) CBC parameters; (3) Heavy metals and trace elements (lead, cadmium, total mercury, selenium, and manganese); (4) Glycohemoglobin (HbA1c); (5) Total cholesterol; and (6) Biochemical biomarkers (eg, sodium, potassium, and alkaline phosphatase).

The principle of this machine learning algorithm (MLA)-based diagnostic depends on the integration of various systemic data points, such as demographic, haematologic, and biochemical, in order to identify non-linear patterns associated with moderate-to-severe periodontitis. This aligns with a broader shift in periodontal research to emphasise the biological underpinnings of disease classification and diagnosis by pointing out how host immune responses and molecular biomarkers can shape periodontal disease activity and progression. Contemporary frameworks increasingly move beyond purely clinical measures toward biologically informed definitions of periodontal disease, incorporating inflammatory, immunological, and microbial signatures to improve disease characterisation and risk assessment.^5^ Within this context, soluble biomarkers derived from saliva, serum, and gingival crevicular fluid have emerged as potential tools for periodontal monitoring and diagnosis, reflecting both local tissue inflammation and systemic immune activation.^7,12,22^ Moreover, emerging evidence shows that nitric oxide–mediated antimicrobial and immunoregulatory pathways, along with circulating cytokine profiles, help in modulating periodontal host–microbe interactions and disease severity.^1,24^ Together, these advances underscore the expanding role of systemic and molecular biomarkers in periodontal diagnostics and support the rationale for exploring non-dental, blood-based indicators as tools for population-level risk stratification.

In this framework, routinely collected laboratory and demographic variables are processed through a gradient-boosted model to generate individualised risk estimates that may support referral decisions in non-dental clinical settings. We hypothesised that an XGBoost classifier leveraging routine systemic biomarkers could effectively screen for moderate-to-severe periodontitis, with environmental toxicants and metabolic markers emerging as key predictive features of these plausible biological associations. The goal of this study was to evaluate the performance of an XGBoost model to predict moderate-to-severe periodontitis using routine blood biomarkers and demographic data. We further assessed subgroup analyses by age and gender to explore disparities in predictive performance. SHAP analysis was used to identify the most influential predictors associated with periodontitis in the general population.

## METHODS AND MATERIALS

### Data Source

**Fig 1:**
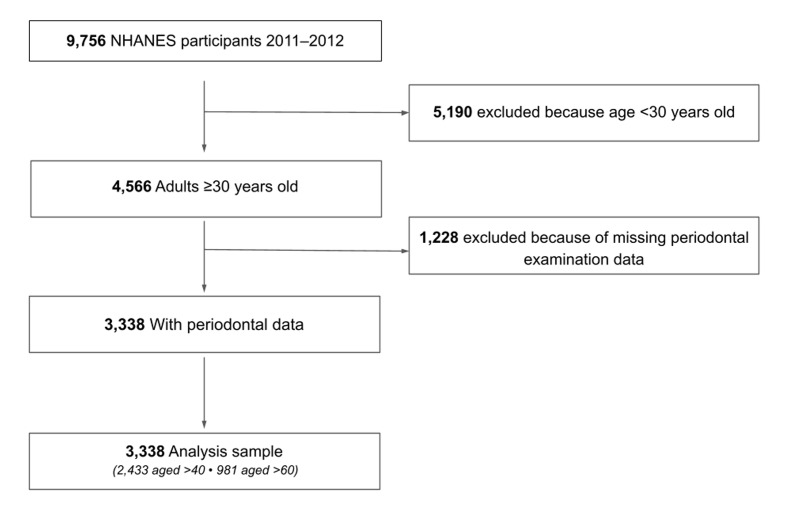
Flowchart of participant selection in NHANES 2011–2012.

This study utilised data from the 2011–2012 cycle of NHANES, a nationally representative US health survey. Periodontal examinations were conducted in NHANES 2011–2012 for adults aged ≥ 30 years. Of 9,756 participants, 4,566 were aged ≥ 30. After excluding 1,228 participants without periodontal data, the final analytic sample included 3,338 individuals (2,433 aged > 40; 981 aged > 60) (Fig 1). Subgroup analyses were performed for those aged > 40 and > 60 years. NHANES data are publicly available, de-identified, and exempt from institutional review.

### Assessment of Periodontal Status

Trained and calibrated dentists conducted full-mouth periodontal examinations in NHANES 2011–2012. Measurements were made at six sites around each tooth using a Hu-Friedy PCP 2 periodontal probe. Periodontitis was defined using the CDC/AAP case definitions, which classify moderate periodontitis as having ≥ 2 interproximal sites with ≥ 4 mm clinical attachment loss (not on the same tooth) or ≥ 2 interproximal sites with probing depth ≥ 5 mm, and severe periodontitis as ≥ 2 interproximal sites with ≥ 6 mm clinical attachment loss and ≥ 1 interproximal site with probing depth ≥ 5 mm.^20^

### Predictor and Outcome Variables

Predictor variables included 77 variables across the following domains:

DEMO (Demographics): age, gender
CBC (Complete blood count with five-part): haemoglobin, white blood cell count, platelet count, and additional haematologic measures
PBCD (Heavy metals): cadmium, lead, mercury, selenium
GHB (glycohemoglobin): HbA1c %
TCHOL: Total cholesterol
BIOPRO (Biochemical markers): sodium, potassium, total protein, alkaline phosphatase (ALP), and additional metabolic markers

Variable labels follow NHANES codes (eg, LBXSGT = γ-glutamyl transferase [GGT], LBXBPB = blood lead, LBXBCD = blood cadmium).

Outcome variables included a binary classification of periodontitis based on the CDC/AAP case definitions described earlier:

0 = None to mild periodontitis
1 = Moderate-to-severe periodontitis

A representative snapshot of the training data set, including selected input features, true periodontal status, and model predictions, is shown in Table 1.

**Table 1:** Example of analytic data set structure. Selected input features, periodontal status, and model predictions


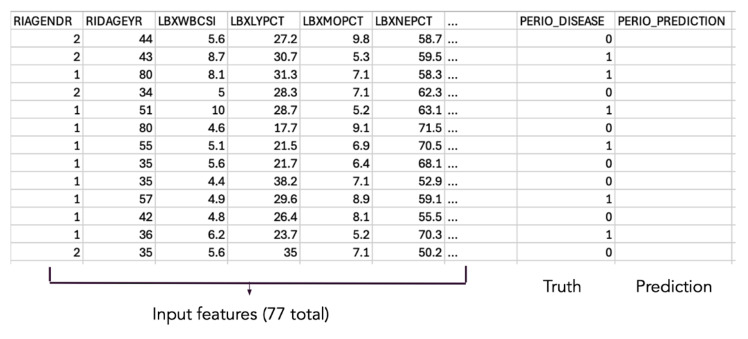


### Model Development

A binary XGBoost classifier was trained using an 80/20 train–test split. Subgroup analyses were conducted by age (> 40 years, > 60 years) and gender.

Rows missing the outcome variable (PERIO_STAGING) were excluded. Predictor variables were merged using left joins; missing predictor values were retained and handled by XGBoost’s built-in missing value mechanism. Gradient boosting models internally assign surrogate splits to manage missingness, avoiding bias introduced by arbitrary imputation and preserving population heterogeneity.

NHANES Mobile Examination Center (MEC) sample weights (WTMEC2YR) were applied during model training to account for the complex survey design and improve population representativeness. Model performance was evaluated on the held-out test set, with Accuracy, Precision, Recall, and F1 score reported. Sensitivity analyses were performed by varying the decision threshold and by re-training the model with and without the application of MEC weights during training. All evaluation metrics were calculated unweighted, consistent with our primary analysis. For all performance metrics, 95% confidence intervals were obtained via bootstrap with 1,000 resamples of the test set. Full threshold diagnostics (precision–recall curve and threshold sweep) are provided in Supplementary Figures S1 and S2.

The source code is available from the corresponding author upon reasonable request.

### Survey Design and Weighting

NHANES uses a complex, multistage probability design with oversampling of specific subpopulations. To improve population representativeness during model fitting, we supplied the Mobile Examination Center 2-year weights (WTMEC2YR) as sample weights to the XGBoost learner. All evaluations of the classifier (Accuracy, Precision, Recall, F1, CIs) were conducted unweighted on the held-out test set, consistent with our primary goal of algorithmic performance assessment rather than population estimation. Descriptive prevalence can be reported with survey weights; however, weighted prevalence estimation was outside the scope of this modelling study. Unless otherwise specified, descriptive counts and proportions reported in the manuscript are based on the analytic sample (unweighted), and all classifier performance metrics are unweighted.

## RESULTS

### Participant Characteristics

Of the 3,338 participants aged ≥ 30 years included, 52.8% were classified as having moderate-to-severe periodontitis. The mean age was 51.82 years (SD: 14.2). The sample was nearly evenly split by gender (49.3% males, 50.7% females). Prevalence was notably higher among males (61.7%) compared to females (44.2%). Periodontitis was associated with elevated glycohaemoglobin (HbA1c) and blood cadmium levels.

### Model Performance

Overall model performance:

Overall, the model achieved 61.1% Accuracy (95% CI: 57.5–64.7), 57.4% Precision (95% CI: 53.4–61.6), 94.6% Recall (95% CI: 92.1–96.8), and an F1 score of 71.5% (95% CI: 68.1–74.7).
Full threshold diagnostics (precision–recall curve and threshold sweep) are provided in Supplementary Figures S1 and S2.

Subgroup analysis by age:

Age > 40: Accuracy = 72.9% (95% CI: 68.9–77.0), Precision = 74.4% (95% CI: 69.9–78.9), Recall = 86.6% (95% CI: 82.6–90.2), and F1 = 80.0% (95% CI: 76.6–83.2).
Age > 60: Accuracy = 68.5% (95% CI: 61.9–75.1), Precision = 69.2% (95% CI: 62.4–75.9), Recall = 96.2% (95% CI: 92.6–99.2), and F1 = 80.5% (95% CI: 75.5–85.1).

Subgroup analysis by gender:

Males: Accuracy = 66.7% (95% CI: 61.3–71.8), Precision = 65.5% (95% CI: 59.9–70.8), Recall = 96.9% (95% CI: 94.2–99.0), and F1 = 78.2% (95% CI: 73.9–82.0).
Females: Accuracy = 56.3% (95% CI: 50.9–61.6), Precision = 48.9% (95% CI: 43.0–54.8), Recall = 92.2% (95% CI: 87.6–96.1), and F1 = 63.9% (95% CI: 58.3–69.2).

Subgroup F1 scores at the chosen operating threshold are summarised in Supplementary Figure S3.

### Feature Importance Analysis

**Fig 2:**
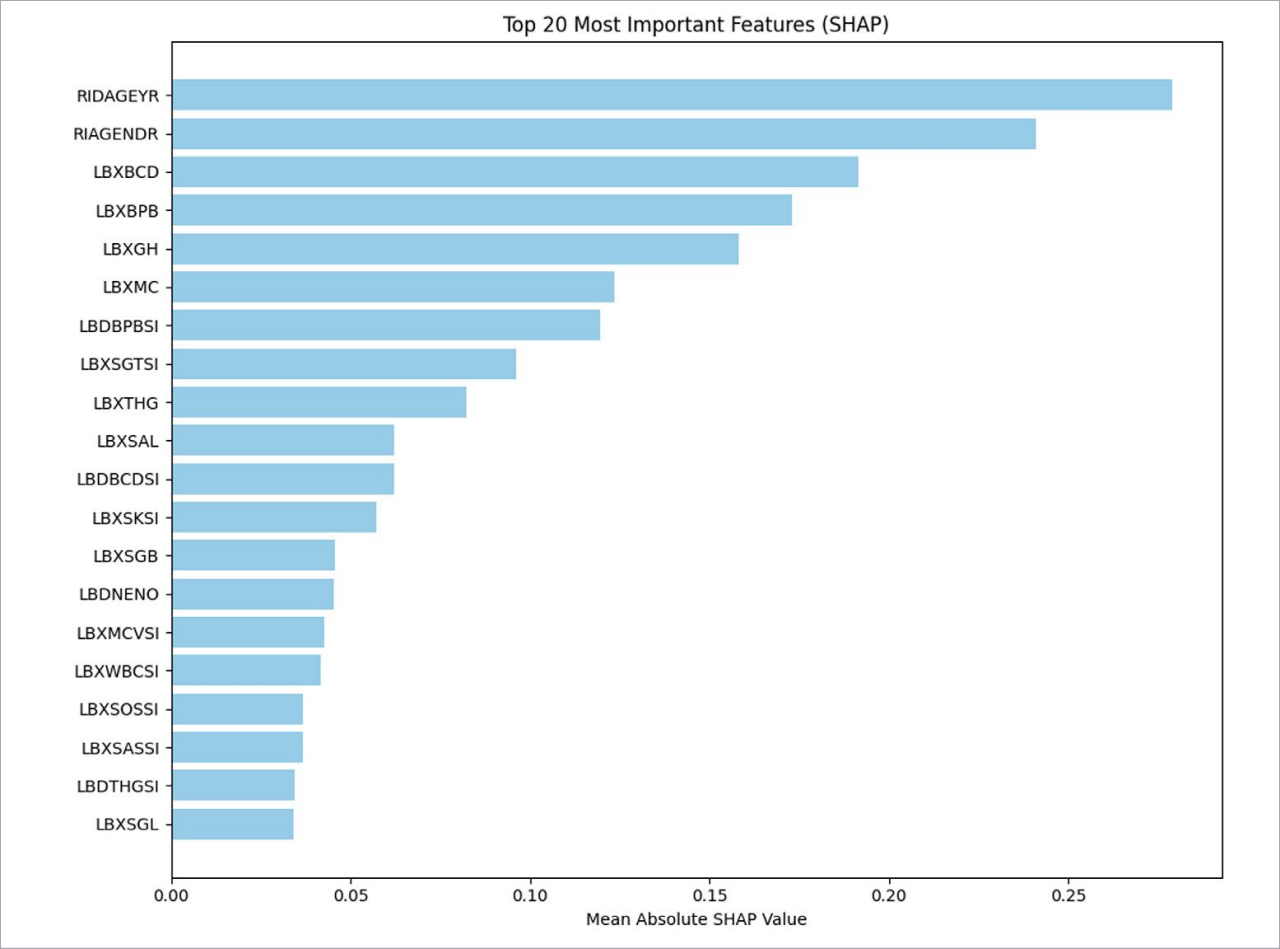
SHAP bar plot of the top 20 most important features ranked by mean absolute SHAP value. Higher SHAP values indicate a stronger contribution to the predicted risk of moderate-to-severe periodontitis.

**Fig 3:**
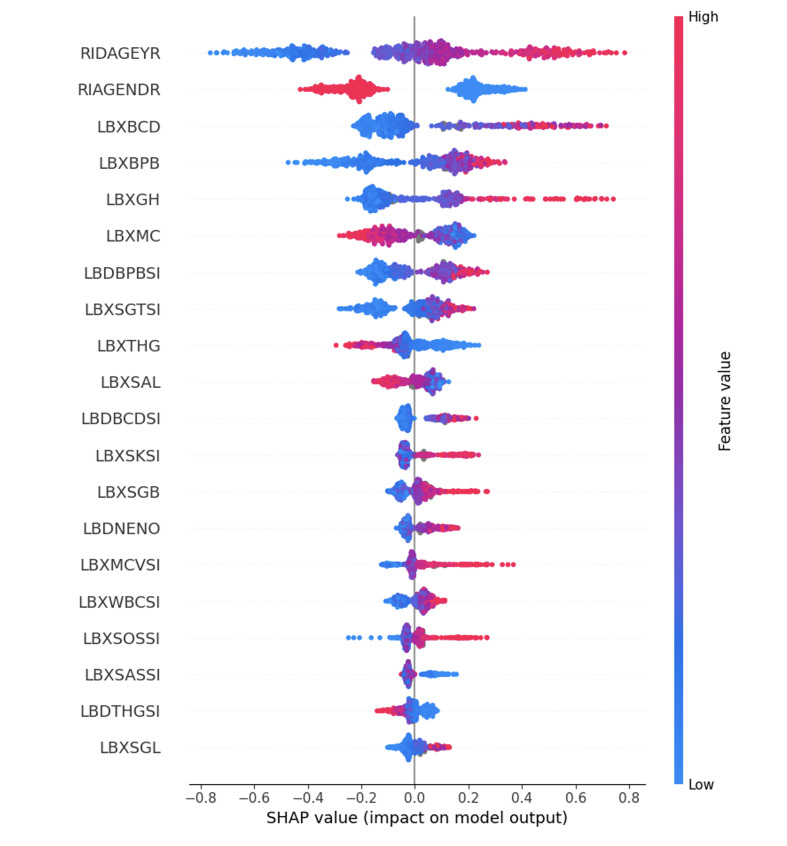
SHAP summary plot of the top predictor features. Each point represents a SHAP value for an individual participant, with colour indicating feature value (red = high, blue = low). Positive SHAP values (> 0) indicate increased predicted risk of moderate-to-severe periodontitis, while negative SHAP values (< 0) indicate decreased predicted risk.

To interpret the model’s predictions, SHapley Additive exPlanations (SHAP) were used to estimate the contribution of each input feature. The top five predictors ranked by mean SHAP value, in order, were age (RIDAGEYR), gender (RIAGENDR), blood cadmium (LBXBCD), blood lead (LBXBPB), and glycohaemoglobin (LBXGH). Refer to Table 2 for the complete list of the top 20 features with definitions and SHAP values, Figure 2 for their ranked magnitudes, and Figure 3 for the directionality of each feature’s effect (high vs low values).

**Table 2:** Top 20 predictive features ranked by mean SHAP value in the final XGBoost model

Rank	Feature label	Feature definition	SHAP value
1	RIDAGEYR	Age (years)	0.279
2	RIAGENDR	Gender (1 = Male, 2 = Female)	0.241
3	LBXBCD	Blood cadmium (µg/L)	0.191
4	LBXBPB	Blood lead (µg/dL)	0.173
5	LBXGH	Glycohemoglobin (%)	0.158
6	LBXMC	Mean corpuscular hemoglobin concentration (g/dL)	0.124
7	LBDBPBSI	Blood lead (µmol/L, SI units)	0.119
8	LBXSGTSI	Gamma-glutamyl transferase (U/L, SI units)	0.096
9	LBXTHG	Blood mercury (µg/L)	0.082
10	LBXSAL	Serum albumin (g/dL)	0.062
11	LBDBCDSI	Blood cadmium (µmol/L, SI units)	0.062
12	LBXSKSI	Serum potassium (mmol/L, SI units)	0.057
13	LBXSGB	Serum globulin (g/dL)	0.046
14	LBDNENO	Segmented neutrophils (absolute number, 1000 cells/µL)	0.045
15	LBXMCVSI	Mean corpuscular volume (fL, SI units)	0.043
16	LBXWBCSI	White blood cell count (10^3^ cells/µL, SI units)	0.042
17	LBXSOSSI	Serum osmolality (mmol/kg, SI units)	0.037
18	LBXSASSI	Serum aspartate aminotransferase (U/L, SI units)	0.037
19	LBDTHGSI	Blood mercury (µmol/L, SI units)	0.034
20	LBXSGL	Serum glucose (mg/dL)	0.034


### Sensitivity Analyses

Varying the classification threshold demonstrated the expected precision–recall trade-off (Supplementary Fig S2). At τ = 0.30, recall was 0.910 and precision was 0.580, whereas at τ = 0.70, precision improved to 0.775 at the expense of recall (0.319). At the F1-optimal threshold (τ = 0.2646), the model achieved Accuracy = 0.613, Precision = 0.575, Recall = 0.949, and F1 = 0.716. Numerical values for selected operating points are reported in Supplementary Table S1.

Training the identical model without MEC weights yielded similar results. The unweighted model achieved its best F1 at τ = 0.4208 (Accuracy = 0.651, Precision = 0.615, Recall = 0.863, F1 = 0.718). When evaluated at the weighted model’s threshold (τ = 0.2646), performance remained comparable (Accuracy = 0.582, Precision = 0.554, Recall = 0.967, F1 = 0.704). These findings indicate stability across decision thresholds and training-weighting schemes, with all evaluations performed unweighted for consistency with the primary analysis. A side-by-side comparison of training with versus without MEC weights is provided in Supplementary Table S2.

### Cluster Analysis

Unsupervised K-means clustering on the top 10 SHAP-ranked features revealed separation of participants into epidemiologically meaningful groups. With k = 2, the model partitioned the test set into two clusters of similar size (n = 315 and n = 336), with moderate-to-severe periodontitis prevalence of 61.6% and 42.0%, respectively. Cluster 0 was characterised by male predominance (RIAGENDR = 1.0), slightly higher blood lead (1.95 µg/dL), and higher γ-glutamyl transferase, whereas Cluster 1 was composed predominantly of females (RIAGENDR = 2.0) with relatively higher cadmium levels (0.64 µg/L). Cluster-level standardised means for the top SHAP features are shown in Supplementary Figure S4.

When k = 3 was specified, an additional very small cluster (n = 1) emerged with extremely heavy metal values (cadmium = 1.80 µg/L, lead = 38.9 µg/dL), which likely represents an outlier rather than a stable subgroup. The other two clusters mirrored the k = 2 solution (Cluster 0: prevalence = 61.8%, Cluster 2: prevalence = 42.0%). Silhouette scores were similar for k = 2 (0.171) and k = 3 (0.173), favouring the more interpretable two-cluster solution. These findings suggest natural separation into higher- and lower-risk subgroups that align with known demographic and biomarker profiles.

### External Context

The prevalence of moderate-to-severe periodontitis in our NHANES 2011–2012 sample was 52.8%. In contrast, studies leveraging the BigMouth Dental Data Repository – a multi-institutional electronic health record (HER) resource from US dental schools – describe clinic-based cohorts with substantially higher periodontal disease burden, reflecting treatment-seeking populations rather than NHANES’s community sampling.^27^ Moreover, CDC small-area modelling indicates marked state-level variation in periodontitis (eg, any periodontitis varying widely across states in 2009–2012), underscoring geographic heterogeneity that differs from national averages.^13^

(Note: we intentionally don’t give a single ‘BigMouth prevalence’ number because it varies by site/definition; the citations characterise BigMouth and point to higher-burden clinical cohorts.)

## DISCUSSION

Our study demonstrates the feasibility of using routine blood test data and demographic variables to screen individuals at risk of moderate-to-severe periodontitis using routinely available medical data. The XGBoost classifier achieved high recall and moderate precision, especially in older adults and males. Feature importance analysis revealed that the most influential predictors were age, gender, blood cadmium, blood lead, and HbA1c – markers consistent with established biological pathways involved in periodontal disease and findings in existing literature.

### Top Predictive Features and Interpretability

Age was the strongest predictor of periodontitis in our model, with a SHAP value of 0.279. This association is well-documented in the literature and can be attributed to several biological and behavioural factors. Ageing is often accompanied by a decline in oral hygiene practices and increased frailty, which can contribute to the progression of periodontal disease.^9^ Biologically, the processes of immunosenescence and inflammaging, referring respectively to the age-related decline in immune function and the presence of chronic low-grade systemic inflammation, further compromise oral immune defences and worsen periodontal tissue breakdown.^11^ Together, these factors help explain why older age is consistently linked to greater periodontitis risk. These effects align with epidemiological data showing increasing severity of periodontitis in older age groups, particularly beyond age 60.^4,23^

Gender was another highly influential demographic predictor (SHAP = 0.241). According to the SHAP summary plot, male gender was positively associated with periodontitis risk, whereas female gender contributed negatively to the prediction. Males are known to have a higher prevalence and severity of periodontitis, likely due to a combination of heightened pro-inflammatory cytokine profiles and hormone-regulated immune responses.^25^ Behavioural factors such as lower oral hygiene adherence and higher smoking rates further exacerbate this risk.^17^ The binary contrast in SHAP contributions underscores the predictive relevance of gender as a categorical variable in periodontal risk modelling.

Blood cadmium (SHAP = 0.191) and lead (SHAP = 0.173) also ranked among the top predictors, reinforcing prior findings that link environmental heavy metal exposure to increased periodontitis risk. Large-scale studies, including NHANES and KNHANES analyses, have consistently shown a positive association between elevated blood or urinary levels of cadmium (Cd) and lead (Pb) and the prevalence of moderate-to-severe periodontitis.^10,16,19,28^ Mechanistically, cadmium and lead contribute to periodontal breakdown through several biological pathways. These metals impair immune function, disrupt bone remodelling, and induce oxidative stress and inflammation in periodontal tissues.^16^ Inflammatory markers such as leukocyte counts have been shown to partially mediate this relationship, suggesting a systemic link between toxic metal exposure and periodontal destruction.^10^ Our findings echo this literature and suggest that cadmium and lead may serve as important systemic indicators of heightened periodontitis risk, particularly in populations with higher environmental exposure.

The identification of blood cadmium and lead as top predictors is biologically plausible. Mechanistically, these metals have been associated with increased oxidative stress, immune dysregulation, and altered bone remodelling pathways, which may contribute to an inflammatory milieu relevant to periodontal disease. Experimental and epidemiologic studies have suggested links between heavy metal exposure and systemic inflammatory responses, which may indirectly relate to periodontal tissue breakdown. Furthermore, it is important to note that elevated heavy metal levels may also serve as proxy indicators for broader environmental or socioeconomic disparities, which independently influence oral health outcomes. Therefore, caution is warranted to avoid overinterpreting these associations purely as direct biological mechanisms. Given the cross-sectional design of the present study, these findings should be interpreted as statistical associations rather than evidence of a causal relationship.

HbA1c emerged as another influential systemic marker with a SHAP value of 0.158, reinforcing the strong, bidirectional link between glycemic control and periodontal disease. Hyperglycaemia promotes the formation of advanced glycation end-products (AGEs), triggering inflammation, collagen breakdown, and impaired tissue repair, all of which contribute to periodontal destruction.^2^ Notably, this association is also shown even in non-diabetic individuals, where elevated HbA1c levels have been linked to increased risk of periodontitis, suggesting early metabolic dysfunction.^3^ Conversely, treating periodontal disease has been shown to improve glycaemic control, reducing HbA1c by 0.3–0.4%.^21^ These findings highlight the value of HbA1c as a systemic marker in predictive models for oral health.

### Evaluation Metrics and Model Performance

Our machine learning model demonstrated strong performance for identifying individuals at risk of moderate-to-severe periodontitis. On the test data, which was not used during training, the XGBoost classifier achieved an F1 of 71.5%, with strong recall (94.6%) and moderate precision (57.4%). This means that nearly 9 out of 10 individuals with disease were correctly flagged by the model (high sensitivity), though about 6 out of 10 flagged individuals truly had disease (precision). In the context of screening, such high recall is especially valuable: false negatives are minimised, making the tool well-suited for identifying at-risk individuals who might otherwise go undetected, particularly in settings where access to dental care is limited. These results demonstrate the feasibility of using routine blood biomarkers and demographic data for non-invasive periodontal disease screening.

Subgroup analysis revealed disparities in model performance by gender. The model performed significantly better in males (F1 = 78.2%, 95% CI: 73.9–82.0) than in females (F1 = 63.9%, 95% CI: 58.3–69.2), suggesting greater predictive alignment with male-associated risk profiles. This finding aligns with previous studies showing that men tend to exhibit more severe periodontal disease, potentially due to both biological and behavioural factors. Elevated inflammatory responses, sex hormone influences, lower oral hygiene adherence, and higher smoking rates in men may collectively increase their periodontal risk and enhance model sensitivity for this group. These biological and lifestyle differences likely contributed to the improved performance in males observed in our study.

### Subgroup Analysis and Disparities

Our subgroup analyses showed improved model performance in older age groups. Among individuals over 40, the model achieved 72.9% Accuracy, 74.4% Precision, 86.6% Recall, and an F1 score of 80.0%, while those over 60 achieved similarly strong metrics (Accuracy 68.5%, Precision 69.2%, Recall 96.2%, F1 score 80.5%). This enhanced performance likely reflects the higher prevalence and severity of periodontitis in older adults, consistent with epidemiological data showing a linear increase in clinical attachment loss and gingival recession with age.^4,23^ Biological processes such as immunosenescence, inflammaging, and frailty may further amplify disease signals and improve model sensitivity in these populations.^9,11^ By contrast, gender-stratified analysis revealed disparities: the model performed better in males (Accuracy 66.7%, F1 78.2%) than in females (Accuracy 56.3%, F1 63.9%). This difference aligns with the higher prevalence of moderate-to-severe periodontitis among males in our data set (61.7% vs 44.2% in females). From an epidemiologic perspective, this may reflect differential misclassification by sex or residual model bias due to class imbalance. Future refinement should incorporate fairness-aware machine learning (ML) approaches, such as stratified sampling or re-weighting to address these disparities.

While these findings support the utility of our model in specific demographic groups, they also inform the necessity of exploring and developing specialised predictive models tailored to different age and gender subpopulations. Future research should prioritise refining these subgroup-specific models to further enhance detection accuracy and clinical utility, particularly in younger and female cohorts where performance was comparatively lower. Additionally, integrating longitudinal data and biomarkers associated with immunosenescence and frailty could highly increase model robustness. Addressing these limitations and expanding subgroup analyses will be crucial for advancing personalised periodontal risk prediction and management strategies.

### Limitations

First, the cross-sectional design limits our ability to infer causality between risk factors and periodontal disease progression. Additionally, potential data imbalance and model bias may have influenced the predictive performance, particularly across different demographic groups. The underlying heterogeneity of risk factors, including biological and behavioural variables, could not be fully accounted for in the model. Furthermore, our analysis was restricted to a single NHANES cycle (2011–2012). Replication across later cycles is necessary to enhance external validity and assess the temporal robustness of the findings, even though weights were applied during training to improve representativeness. We were unable to evaluate model performance by US state or census region because subnational geocodes are restricted in public NHANES files; consequently, we could not assess geographic heterogeneity or calibrate thresholds for specific regions/states, which may limit generalizability where local disease burden differs.^13^ These limitations reinforce that our model should be interpreted as a population-level screening and prevention aid, rather than a diagnostic tool.

Our operating threshold (τ = 0.2646) was chosen post hoc to maximise F1 on the held-out test set. Because the same data were used to select τ and to evaluate performance, the reported point estimates may be modestly optimistic. We partially address this by providing threshold-sensitivity plots (Supplementary Figs S1 and S2) and a weighted vs unweighted training comparison (Supplementary Table S2), but future work will pre-specify τ (eg, via cross-validation) and reserve an external test set for final reporting.

### Epidemiologic Biases

Our analysis is subject to inherent epidemiologic biases associated with the NHANES design. First, selection bias may limit generalizability, as NHANES excludes institutionalised individuals and relies on voluntary participation, which may under-represent those with severe systemic illness or advanced periodontitis. This ‘healthy participant’ effect could lead to an underestimation of true disease prevalence.

Second, misclassification bias is possible. Although periodontal assessments were performed by trained and calibrated examiners, probing measurements are operator-dependent and subject to intra- and inter-examiner variability. Furthermore, the CDC/AAP case definitions used in this study, while standardised, may misclassify borderline cases or fail to capture early disease.

Third, confounding remains a limitation. Important behavioural and environmental variables such as oral hygiene practices, smoking intensity, and socioeconomic status may not have been fully accounted for in the model despite their known associations with periodontal disease. This unmeasured confounding could influence both biomarker distributions and periodontal outcomes.

To mitigate these biases, we incorporated NHANES MEC sample weights to improve representativeness, employed standardised case definitions, and conducted subgroup analyses to assess consistency across demographic groups. Nevertheless, these sources of bias highlight the need for cautious interpretation and underscore that our model is best viewed as a population-level screening tool rather than a diagnostic instrument.

Sensitivity analyses demonstrated that model performance was stable across reasonable thresholds and whether MEC weights were applied during training. Since all evaluations were conducted unweighted, consistent with our primary analysis, these findings underscore that our results are not artefacts of survey weighting or threshold choice. This supports the reliability of the model as a flexible screening aid rather than a fixed diagnostic classifier.

Cluster analysis confirmed that participants grouped into subpopulations with differing prevalence of periodontitis, primarily distinguished by gender and biomarker patterns. This supports the epidemiologic validity of the model and highlights the potential of cluster-informed approaches to mitigate bias and tailor screening thresholds to specific subgroups.

### Clinical Implications

This model is intended as a screening aid for risk stratification rather than a diagnostic instrument. The NHANES periodontal exam data set does not provide sufficiently detailed data for comprehensive periodontal assessment and diagnosis, which limits its clinical depth. It may, however, aid in flagging individuals at risk who might not seek dental care but undergo routine medical testing. Integration into primary care settings or electronic health record systems could prompt referrals for periodontal evaluation. Especially in underserved populations, this approach may bridge gaps in oral healthcare access and support preventive strategies by linking medical and dental care. Similar to established risk calculators used in primary care, such as the ASCVD (Atherosclerotic Cardiovascular Disease) risk estimator for cardiovascular disease, this model is designed to provide a probabilistic risk score to guide referrals rather than a definitive clinical diagnosis.

This approach would enable primary care providers to better act early and risk-stratify patients, addressing bidirectional oral-systemic risks earlier, particularly the well-established interplay between periodontitis and diabetes, where poor glycemic control (eg, elevated HbA1c) exacerbates periodontal inflammation and destruction, while untreated periodontitis impairs insulin action and worsens glycemic outcomes through systemic microinflammation. Ultimately, it supports more proactive, interdisciplinary management of patients at elevated risk for periodontal disease, fostering timely referrals, integrated care pathways, and improved overall health through collaborative efforts between primary care, endocrinology, and periodontal specialists.

### Future Directions

Given these findings, future work should focus on developing specialised models using stratified sampling approaches to better tailor predictions for distinct age and gender groups. Further training, refinement, and validation of these subgroup-specific models are essential to enhance accuracy and clinical relevance, with a focus on younger individuals and females, where performance was comparatively lower. Expanding model inputs to include biomarkers of ageing and frailty and conducting longitudinal analyses will also be important next steps. Future work will pursue restricted geocode access (eg, through the NCHS Research Data Center) and external clinical data sets to quantify regional/state performance and, if needed, tune thresholds for local deployment. Ultimately, these efforts aim to advance personalised periodontal disease risk prediction and inform targeted prevention and treatment strategies.

## CONCLUSION

Our study demonstrates the feasibility of using routine blood tests and demographic data to identify individuals at risk for moderate-to-severe periodontitis using machine learning. The XGBoost classifier achieved high recall and moderate precision, particularly among older adults and males. Feature importance analysis highlighted age, gender, blood cadmium, blood lead, and HbA1c as the top predictors – markers consistent with known biological mechanisms of periodontal disease and existing literature.

### Acknowledgements

#### Ethics approval and consent to participate

The NHANES protocol was approved by the National Center for Health Statistics Research Ethics Review Board. Written informed consent was obtained from all participants. This analysis used publicly available, de-identified data and was exempt from further institutional review.

#### Reporting guidelines

This study followed the STROBE (Strengthening the Reporting of Observational Studies in Epidemiology) reporting guideline for cross-sectional studies.

#### Clinical trial number

Not applicable.

#### Consent for publication

Not applicable.

#### Availability of data and materials

The NHANES 2011–2012 data sets analysed in this study are publicly available from the Centers for Disease Control and Prevention (CDC): https://wwwn.cdc.gov/nchs/nhanes/continuousnhanes/overview.aspx?BeginYear=2011

The source code is available from the corresponding author upon reasonable request.

#### Competing interests

The authors declare that they have no competing interests.

#### Funding

This research received no external funding.

## References

[ref1] Ahmad P, Slots J, Siqueira WL (2025). Serum cytokines in periodontal diseases. Periodontol 2000.

[ref3] Banjar A, Alyafi R, AlGhamdi A, Assaggaf M, Almarghlani A, Hassan S, Mealey B (2023). The relationship between glycated hemoglobin level and the stage of periodontitis in individuals without diabetes. PLoS One.

[ref6] Botelho J, Machado V, Hussain SB, Zehra SA, Proença L, Orlandi M (2021). Periodontitis and circulating blood cell profiles: a systematic review and meta-analysis. Exp Hematol.

[ref7] Buduneli N, Bıyıkoğlu B, Kinane DF (2024). Utility of gingival crevicular fluid components for periodontal diagnosis. Periodontol 2000.

[ref9] Clark D, Kotronia E, Ramsay SE (2021). Frailty, aging, and periodontal disease: basic biological considerations. Periodontol 2000.

[ref10] Du M, Deng K, Cai Q, Hu S, Chen Y, Xu S (2024). Mediating role of systemic inflammation in the association between heavy metals exposure and periodontitis risk. J Periodontol.

[ref11] Ebersole JL, Dawson D 3rd, Emecen-Huja P, Nagarajan R, Howard K, Grady ME (2017). The periodontal war: microbes and immunity. Periodontol 2000.

[ref12] Ebersole JL, Hasturk H, Huber M, Gellibolian R, Markaryan A, Zhang XD, Miller CS (2024). Realizing the clinical utility of saliva for monitoring oral diseases. Periodontol 2000.

[ref13] Eke PI, Zhang X, Lu H, Wei L, Thornton-Evans G, Greenlund KJ (2016). Predicting periodontitis at state and local levels in the United States. J Dent Res.

[ref14] Eley BM, Cox SW (1998). Advances in periodontal diagnosis. 1. Traditional clinical methods of diagnosis. Br Dent J.

[ref15] Grover HS, Luthra S (2013). Molecular mechanisms involved in the bidirectional relationship between diabetes mellitus and periodontal disease. J Indian Soc Periodontol.

[ref16] Han DH, Lim SY, Sun BC, Paek D, Kim HD (2010). The association of metabolic syndrome with periodontal disease is confounded by age and smoking in a Korean population: the Shiwha–Banwol Environmental Health Study. J Clin Periodontol.

[ref17] Ioannidou E (2017). The sex and gender intersection in chronic periodontitis. Front Public Health.

[ref19] Liu Y, Wu Y, Shi X, Tian Y, Zhai S, Yang Z, Chu S (2024). Association between blood lead and periodontitis among American adults: a cross-sectional study of the National Health and Nutrition Examination Survey. Front Pharmacol.

[ref21] Preshaw PM, Bissett SM (2019). Periodontitis and diabetes. Br Dent J.

[ref22] Ramseier CA (2024). Diagnostic measures for monitoring and follow-up in periodontology and implant dentistry. Periodontol 2000.

[ref23] Relvas M, López-Jarana P, Monteiro L, Pacheco JJ, Braga AC, Salazar F (2022). Study of prevalence, severity and risk factors of periodontal disease in a Portuguese population. J Clin Med.

[ref25] Shiau HJ, Reynolds MA (2010). Sex differences in destructive periodontal disease: exploring the biologic basis. J Periodontol.

[ref26] Treloar T, Bishop SS, Dodd V, Shaddox LM (2021). Evaluating true barriers to dental care for patients with periodontal disease. Int J Dent Oral Health.

[ref27] Walji MF, Kalenderian E, Stark PC, White JM, Kookal KK, Phan D (2014). BigMouth: a multi-institutional dental data repository. J Am Med Inform Assoc.

[ref28] Won YS, Kim JH, Kim YS, Bae KH (2013). Association of internal exposure of cadmium and lead with periodontal disease: a study of the Fourth Korean National Health and Nutrition Examination Survey. J Clin Periodontol.

